# Quality of life and clinical outcomes of operatively treated patients with flail chest injuries: A multicentre prospective cohort study

**DOI:** 10.3389/fsurg.2023.1156489

**Published:** 2023-03-15

**Authors:** Ruben J. Hoepelman, Fabrizio Minervini, Frank J. P. Beeres, Bas van Wageningen, Frank F. IJpma, Nicole M. van Veelen, Koen W. W. Lansink, Jochem M. Hoogendoorn, Mark. C. P. van Baal, Rolf H. H. Groenwold, Roderick M. Houwert, on behalf of the NEXT study group

**Affiliations:** ^1^ Department of Trauma Surgery, University Medical Center Utrecht, Utrecht, Netherlands; ^2^ Department of Thoracic Surgery, Luzerner Kantonsspital, Lucerne, Switzerland; ^3^ Department of Orthopedics and Trauma Surgery, Luzerner Kantonsspital, Lucerne, Switzerland; ^4^ Department of Trauma Surgery, Radboud University Medical Center Utrecht, Nijmegen, Netherlands; ^5^ Department of Trauma Surgery, University Medical Center Groningen, Groningen, Netherlands; ^6^ Department of Trauma Surgery, Elisabeth-Tweesteden Hospital, Tilburg, Netherlands; ^7^ Department of Trauma Surgery, Haaglanden Medical Center, Hague, Netherlands; ^8^ Department of Clinical Epidemiology, Leiden University Medical Center, Leiden, Netherlands; ^9^ Department of Biomedical Data Sciences, Leiden University Medical Center, Leiden, Netherlands

**Keywords:** rib fixation, flail chest, rib fracture, quality of life, thoracic trauma

## Abstract

**Introduction:**

Most studies about rib fractures focus on mortality and morbidity. Literature is scarce on long term and quality of life (QoL) outcomes. Therefore, we report QoL and long-term outcomes after rib fixation in flail chest patients.

**Methods:**

A prospective cohort study of clinical flail chest patients admitted to six level 1 trauma centres in the Netherlands and Switzerland between January 2018 and March 2021. Outcomes included in-hospital outcomes and long-term outcomes, such as QoL measurements 12 months after hospitalization using the EuroQoL five dimensions (EQ-5D) questionnaire.

**Results:**

Sixty-one operatively treated flail chest patients were included. Median hospital length of stay was 15 days and intensive care length of stay was 8 days. Sixteen (26%) patients developed pneumonia and two (3%) died. One year after hospitalization the mean EQ5D score was 0.78. Complication rates were low and included hemothorax (6%) pleural effusion (5%) and two revisions of the implant (3%). Implant related irritation was commonly reported by patients (*n* = 15, 25%).

**Conclusions:**

Rib fixation for flail chest injuries can be considered a safe procedure and with low mortality rates. Future studies should focus on quality of life rather than solely short-term outcomes.

**Trial registration:** Registered in the Netherlands Trial Register NTR6833 on 13/11/2017 and the Swiss Ethics Committees Registration Number 2019-00668

## Introduction

Thoracic trauma is a potentially life-threatening injury which often result in rib fractures (1). Patients with rib fractures represent a heterogenous group of patients among whom injury severity, morbidity, and mortality differ significantly (2). Distinction between patients with and without a flail chest is paramount, as flail chest injuries are associated with higher morbidity and mortality (1, 2).

Historically, patients with rib fractures were treated nonoperatively, however, since the introduction of the dedicated plating systems, rib fixation has become a routinely performed procedure in many trauma centers (3). Indications for rib fixation include flail chest injuries, failure to wean from mechanical ventilation, severe thorax deformity and inadequately manageable pain through conservative measures. Although rib fixation has not proven beneficial in all patients with multiple rib fractures, it has shown to improve in-hospital outcomes in flail chest patients (2, 4–6). The essence of rib fixation is creating a stable chest wall in order to facilitate adequate ventilation and reduce fracture related pain, caused by paradoxical movement of the chest wall. There are several different methods for rib fixation of which plate osteosynthesis is most widely used (7).

Rib fixation should be performed as soon as possible (preferable within 72 h) after admission, however treatment strategy and timing should still be evaluated for each individual patient, as flail chest patients often suffer from serious intra-and extra-thoracic injuries as well (8).

Mortality after multiple rib fractures has steadily declined in the last decades (1, 9). This is mainly due to the vast improvements in trauma care and intensive care management in general. Therefore, focus in trauma research has shifted from mortality to morbidity and Quality of Life (QoL) (10, 11). Nonetheless, most studies on rib fixation in flail chest patients are still focused on in-hospital outcomes, while studies reporting long term outcomes and quality of life are limited. Therefore, the aim of this study was to report short and long-term outcomes of a cohort of operatively treated flail chest patients.

## Methods

In this study we focus solely on patients with flail chest injuries who received operative treatment. This study adhered to the Strengthening the Reporting of Observational Studies in Epidemiology (STROBE) guidelines (12).

### Patients and study design

This was a multicentre prospective cohort study conducted in five level 1 trauma centres in the Netherlands and one in Switzerland. All patients 18 years and older with computerized tomography (CT) scan confirmed flail chest injuries, which is defined as clinically visible paradoxical movement of a portion of the chest wall and three or more consecutive ribs broken, each in at least two places, were eligible for inclusion. Patients were excluded in case of cognitive impairment, non-traumatic rib fractures, and rib fractures due to cardiopulmonary resuscitation. The study was registered in the Netherlands Trial Registry (NTR6833). Approval from the institutional review board was obtained at every study site. Informed consent was obtained from all participants.

### Rib fixation

Rib fixation was always performed or supervised by a senior thoracic and/or orthopaedic trauma surgeon with experience in surgical treatment of rib fractures. Preoperative planning was done using a chest CT scan with 3D reconstruction. Prophylactic preoperative antibiotic therapy (2 g Cefazolin i.v.) was administered to all patients. The surgical approaches were performed as described by Taylor et al. (6). After reduction, ideally internal fixation was performed using at least three bicortical screws on each side of the fracture and the MatrixRIB system (Depuy Synthes). The number of ribs fixated was left to the discretion of the attending surgeon, with the goal of achieving a stable thorax. Consequently, not all fractured ribs associated with the flail segment were always stabilized.

### Patient characteristics

Patient characteristics measured at hospital admission included age, sex, body mass index (BMI), American Society of Anaesthesiologist (ASA) score, presence of chronic obstructive pulmonary disease (COPD), smoking status, trauma mechanism, abbreviated injury scale (AIS) score (13), injury severity score (ISS), number of fractured ribs, concomitant injuries (i.e., pulmonary contusion, pneumothorax, haemothorax, sternum, and/or clavicle fracture), and laboratory results (specifically venous blood pH and base excess) (14, 15).

### Outcomes

Outcomes included: Intensive care unit length of stay (ILOS), hospital length of stay (HLOS), duration of mechanical ventilation (DMV), need for tracheostomy, pneumonia rate and other in-hospital complications, in-hospital mortality rate, and general pain [measured using a numeric rating scale (NRS)]. Mid- and long-term outcomes were measured at the outpatient clinic visit at 6 weeks and using telephone interviews after 12 months. These measures included pain with breathing and coughing (measured using the NRS), quality of life (measured using the EQ5D), dyspnoea burden [measured using the modified Medical Research Council (mMRC) dyspnoea scale], and return to work and sports in weeks. Surgery-specific complications included fracture-related infection, symptomatic non-union, and implant removal (assessed using Hulsmans et al.'s algorithm) (16, 17). Pneumonia was defined as clinical signs or symptoms (two or more present; temperature > 38.5°C, auscultation with suspicion for infiltrate, or purulent sputum) and/or additional tests (thoracic radiographs with signs of infiltrate, leucocytosis, elevated C-reactive protein,) for which antimicrobial therapy was needed. Acute respiratory distress syndrome (ARDS) was defined according to the Berlin definition (18). Fracture-related infections were diagnosed according to the FRI consensus definition (16). Symptomatic non-union was defined as the presence of unsuccessfully healed ribs, confirmed by CT scan, at least 6 months after trauma, with clinical evidence of pain. The EQ5D-5L is a standardized instrument for generic health status measurements to assess the quality of life (19). The mMRC is a five-category scale that characterizes the level of dyspnoea with physical activity (20).

### Statistical analyses

Data were analyzed using SPSS version 28.0 (SPSS, Chicago, USA). Missing values were not imputed. Continuous variables were presented as mean with standard deviation or median with interquartile ranges (IQR). Categorical data were presented as frequencies and percentages. A one-sample T-test was used to compare the EQ5D index score to the population norm (0.84) (21).

## Results

### Patients

**Figure 1:**
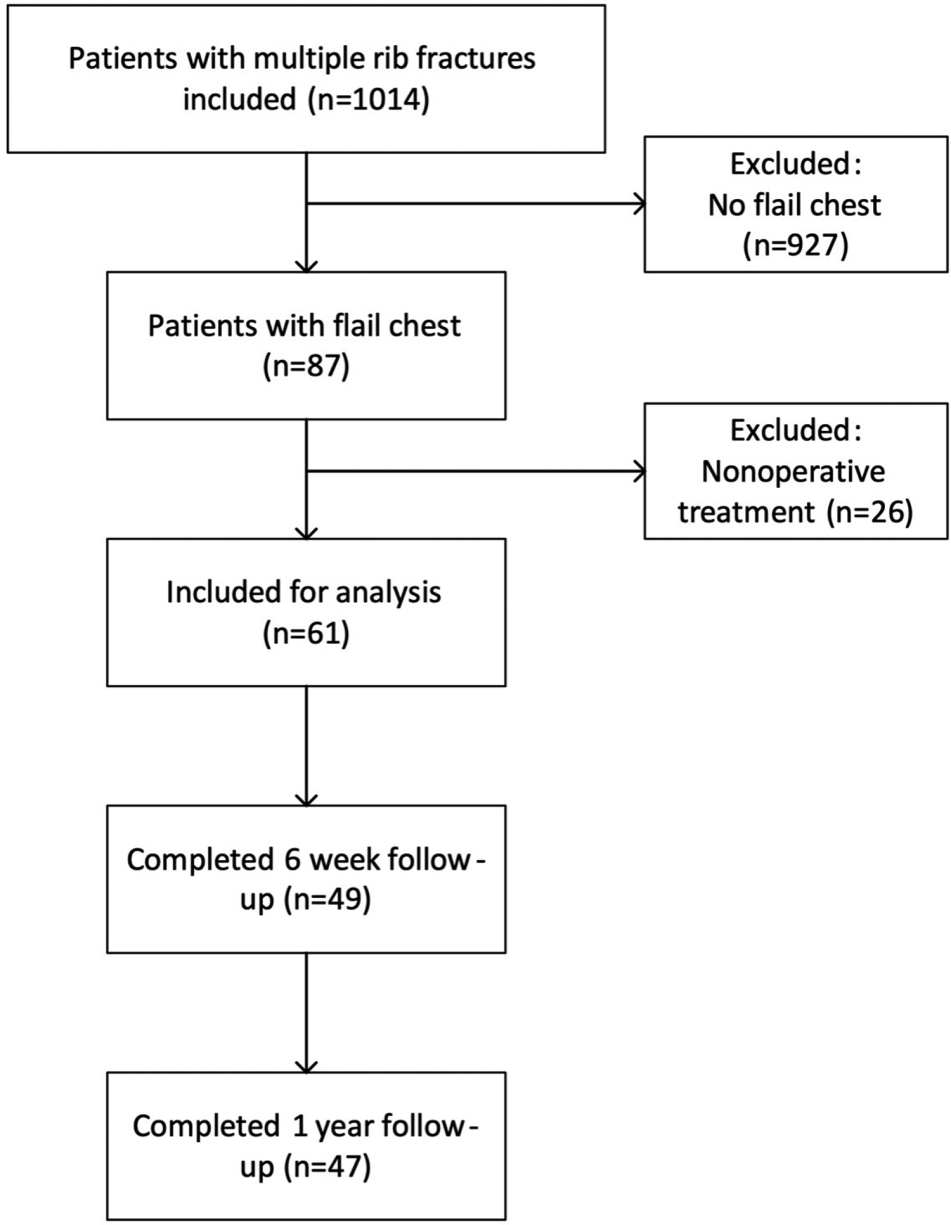
Flow diagram of inclusion and follow-up.

From January 2018 to March 2021, 87 patients with clinical flail chest injuries were included of whom 61 were treated operatively. Follow-up was completed March 2022, with a completion rate of 77% (Figure 1). Mean age was 60.8 ± 15.9, 43 patients (71%) were male and 67% of patients were classified as ASA 1 or 2. Most patients were polytrauma patients with a mean ISS of 26.4 ± 11.9 and a mean Thorax trauma severity score (TTSS) of 12.8 ± 3.5. The median number of rib fractures was 10 (8–12.5), and 52.5% had bilateral fractures. The rate of concomitant thoracic injuries was high; 91.8% had a pneumothorax, 60.7% a haemothorax and 57.4% a pulmonary contusion (Table 1).

**Table 1:** Baseline characteristics of operatively treated patients with flail chest injuries.

Variable	Rib fixation (*n* = 61)
Age (mean ± SD)	60.8 ± 15.9
Male (*n*, %)	43 (70.5)
**ASA-score (*n*, %)**	
1–2	41 (67)
>2	20 (33)
BMI (mean ± SD)	26.1 ± 5.0
COPD, (*n*, %)	7 (12)
Current smoker (*n*, %)	17 (29)
Trauma mechanism (*n*, %)	
Motor vehicle accident	33 (54)
Fall from height/stairs	19 (31)
Other	9 (15)
ISS (mean ± SD)	26.4 ± 11.9)
TTSS (mean ± SD)	12.8 ± 3.5
AIS (median, IQR)	
Head	0 (0–2)
Face	0 (0–.25)
Thorax	4 (3–4)
Abdomen	0 (0–2)
Extremities	2 (0–3)
No. of rib fractures (median, IQR)	10 (8–12.5)
Bilateral rib fractures (*n*, %)	32 (52.5)
**Concomitant thoracic injuries (*n*, %)**	
Pulmonary contusion (*n*, %)	35 (57)
Pneumothorax (*n*, %)	56 (92)
Hemothorax (*n*, %)	37 (61)
Sternum fracture (*n*, %)	10 (16)
Clavicle fracture (*n*, %)	12 (20)
Venous blood pH (mean ± SD)	7.3 ± 0.1
Base excess (mean ± SD)	−3.1 ± 5.5
**Surgery related variables**	
Days until rib fixation (median, IQR)	2 (1–4)
Duration of surgery in minutes (mean ± SD)	143 ± 84
Number of surgically-fixated rib fractures (median, IQR)	4 (3–5)
Ratio of surgically-fixated ribs and total number of rib fractures	.43
Thoracotomy (*n*, %)	14 (23)
Video assisted thoracic surgery (*n*, %)	6 (10)
**Surgical approach (*n*, %)**	
Anterolateral	3 (5)
Lateral	11 (18)
Posterolateral	20 (34)
Posterior	9 (15)
Combination	17 (28)

### Surgery related information

The median number of days to surgery was 2 (1–4) days with a mean duration of surgery of 143 ± 84 min. The median number of fixated ribs was 4 (3–5), which was a proportion of 0.43 of the number of fractured ribs. Video assisted thoracic surgery was performed in 6 patients (10%) aiming to better localize the fractures or identify/treat other thoracic injuries. In total there were two implant related complications (3.3%) for which revisions were performed. One implant dislocated during the initial in-patient stay, while the second dislocation was detected during follow-up. There were no infections nor symptomatic non-unions.

### Primary and secondary outcomes

The median hospital length of stay was 15 (10–28) days. Fourtyseven (77%) patients were admitted to the ICU with a median duration of ICU stay of 8 (3–14) days. Mechanical ventilation was necessary in 38 patients (62%) with a median duration of 6.5 days. Sixteen patient (26%) developed pneumonia; the rate of other (respiratory) complications was low. Two patients died during admission (3.3%), one patient developed pneumonia and died due to respiratory failure. The second patient died due to unrelated causes. All outcomes are available in Table 2.

**Table 2:** Outcomes of operatively treated patients with flail chest injuries.

In-hospital outcomes, median (IQR)	Rib fixation (*n* = 61)	In-hospital complications (*n*, %)	Rib fixation (*n* = 61)
Hospital length of stay	15 (10–28)	ARDS	0 (0)
Hospital length of stay from RF	13 (8–26)	Tracheostomy	5 (8.2)
Need for ICU (*n*, %)	47 (77)	Pneumonia	16 (26.2)
ICU length of stay	8 (3–14)	Pleural effusion	3 (4.9)
Need for ventilation (*n*, %)	38 (62.3)	Pneumothorax	0 (0)
Duration of Invasive mechanical ventilation	6.5 (3–9)	Hemothorax	4 (6.6)
Epidural treatment (*n*, %)	19 (31.1)	Other complication	23 (37.7)
Duration of epidural analgesia	5 (3–7)	Mortality	2 (3.3)
Duration of Intravenous analgesia	8 (3.5–14)	Revision of implant	1 (1.6)
**NRS (pain)**			
Day 3	2 (0–3)		
Day 5	1 (0–3)		
Day 7	1.5 (0–3)		
Mid- and long-term outcomes	Rib fixation (*n* = 61)		Rib fixation (*n* = 61)
Follow up 6 weeks		Follow up 1 year	
EQ5D-5L index value, median (IQR)	0.71 (0.58–0.80)	EQ5D-5l index value, median (IQR)	0.78 (0.57–0.90)
EQ5D-5L VAS, median (IQR)	70 (40–75)	EQ5D-5l VAS, median (IQR)	70 (40–85)
MMRC, median (IQR)	1 (0–2)	MMRC, median (IQR)	0 (0–1)
NRS (pain)		NRS (pain)	
General	3 (1–4)	General	1 (0–5)
Breathing	1 (0–3)	Breathing	0 (0–0)
Coughing	2 (.25–4)	Coughing	0 (0–0)
Complications (*n*, %)		Complications (*n*, %)	
Pneumonia	2 (3.3)	Implant related irritation (*n*, %)	15 (24,6)
Pleural effusion	2 (3.3)	Implant removal (*n*, %)	2 (3.3)
Pneumothorax	0 (0)	Symptomatic non-union (*n*, %)	0 (0)
Hemothorax	0 (0)	Deceased (*n*, %)	1 (1.6)
Fracture related infection	0 (0)	Return to work (weeks), median (IQR)	14 (6.5–19.5)
Dislocated implant	1 (1.6)	Return to sports (weeks), median (IQR)	23 (9.75–32.5)

ARDS, acute respiratory distress syndrome; ICU, intensive care unit; IQR, interquartile range; MMRC, modified medical research council dyspnea scale; NRS, numeric rating scale; RF, rib fixation.

### Mid- and long-term outcomes

Quality of life measured using the EQ5D score was median 0.71 (0.58–0.80) after six weeks and 0.78 (0.57–0.90) after one year, while the median EQ5D VAS score was 70 (40–70) at the six week follow-up and 70 (60–75) at one year. The complication rate was low at six weeks (pneumonia 3% (*n* = 2), pleural effusion 3% (*n* = 2)), while implant irritation after one year was high; 15 patients (24.6%) reported irritation and two patients (3.3%) had the implants removed prior to the one year follow-up. One patient had died prior to the one year follow-up due to causes unrelated to the thoracic trauma. Median return to work was 14 (6.5–19.5) weeks and return to sports was 23 (9.75–32.5) weeks among patients that performed these activities before injury.

## Discussion

This study describes the clinical and long-term outcomes of a prospective cohort of operatively treated flail chest patients. Although all patients were severely injured, mortality rate (3%) and surgery-related complications (3%) were low. Quality of life as measured by the EQ5D was 0.78 (0.6–0.9) after one year and 26% of the patients experienced implant related irritation.

### Comparison to previous literature

There are several previously published prospective studies and trials that report on flail chest patients (22–28). Although comparison of studies is complicated by heterogeneity in inclusion criteria and different definitions of flail chests, most studies report benefits after rib fixation compared to nonoperative treatment in flail chest patients. Advantages are mostly observed in patient who were ventilated at the time of rib fixation.

The results of our study closely resemble those of the operative group of the most recently published RCT on the subject (23). Patients' characteristics and outcomes are remarkably similar, most likely due to similar indications for rib fixation. They conclude a potential advantage with operative treatment in the subgroup of patients who were ventilated at the time of randomization, but not in those that were not ventilated.

Our study adds to current literature as one of the few studies to report 1-year outcomes. Most studies focus on in-hospital or short-term outcomes, which is also demonstrated by Supplementary Material Table S1. Marasco et al. reports QoL after 6 months (21). They reported no difference between operative and nonoperative treatment in SF-36 scores (21), although the reported scores were lower compared to population's norms. Caragounis et al. reports an excellent EQ5D score of 0.93 after rib fixation (28). It is difficult to compare this to our or other studies as they give limited information about their cohort and no in-hospital information. Walters et al. report QoL as well, with varying time to follow-up (mean 17.6 SD 9.5 months) (25). They did not find any differences between their groups, however, interestingly their EQ5D-5l scores are much lower compared to our cohort (0.66 vs. 0.78). It is not readily apparent why this is the case. EQ5D population norms are generally lower in the UK compared to the Netherlands and Switzerland and their reported return rate was 65% (*n* = 36) for the operative group which makes it more susceptible to selection bias compared to the return rate of 77% (*n* = 48) in our cohort (29). TheEQ5D value of 0.78 in our study is also significantly (*p* < 0.001) lower than population norms in the Netherlands (21). However, this is common after trauma and whether this is a clinically important difference remains up to debate (11, 30).

We do consider rib fixation a safe procedure. There were no symptomatic non-unions nor infections and only two (3.3%) implant related complications for which additional surgery was required. These low rates are supported by literature, with complication rates between 1.3–3% (31, 32). Overall surgery related complication rates lie between 10%–16%, but most being minor and not requiring immediate reinterventions (31, 33). Similar good outcomes were reported by De Palma et al. in a retrospective analysis which included 27 patients who underwent surgical stabilization of the thoracic wall for traumatic and non traumatic chest wall disease (34).

Finally, implant irritation is not often reported in studies, but can be a burden to patients. In our study the rate was 26%. Implant irritation predisposes a patient to implant removal. Interestingly, previous research from one of our participating hospitals found plate related irritation rates 53% (33). Whether this decrease is due to more experience (as these results from the prior study were from 6 to 10 years ago), different plating systems or other reasons is unknown. When deciding which patients to operate both the low complication rate and the high implant irritation rate should be kept in mind, as preventing a secondary operation is not only cost effective it also improves trauma patient care.

### Limitations

Several limitations should be acknowledged. The noncomparative design of our study doesn't allow for comparison to other treatment methods (notably nonoperative treatment). However, our study adds to current literature in reporting prospectively gathered long-term outcomes, which are scarce. Second, our follow up does not allow to accurately measure long-term implant removal rate, as follow-up was limited to one year.

Third, EQ5D-5L and mMRC are subjective questionnaires and assess general health and not specifically thorax-related problems. The vast majority of the patients described in this cohort were polytrauma patients; therefore, concomitant injuries but also comorbidities could have influenced the outcome. Lastly, the transition from hospital to home is a critically vulnerable period for patients, which is also dependent on health care system and social class of patients. As there is limited literature on this transition period among trauma patients we do not know how this could have influenced for instance quality of life in our population. For future studies, applying a biopsychosocial model might be able to provide more insight into the matter.

## Conclusion

Flail chest remains a severe injury, however, mortality has declined spectacularly compared to a decade ago. Rib fixation can be considered a safe procedure, but the injury and treatment still impact rehabilitation and quality of life of the patient. Future studies should therefore shift their focus to these aspects to further improve trauma patients care.

## Data Availability

The raw data supporting the conclusions of this article will be made available by the authors, without undue reservation.
